# Evaluation of Physicochemical Properties of a Hydrocolloid-Based Functional Food Fortified with *Caulerpa lentillifera*: A D-Optimal Design Approach

**DOI:** 10.3390/gels9070531

**Published:** 2023-06-29

**Authors:** Nor Atikah Husna Ahmad Nasir, Mohd Hafis Yuswan, Nor Nadiah Abd Karim Shah, Aswir Abd Rashed, Kazunori Kadota, Yus Aniza Yusof

**Affiliations:** 1 Laboratory of Halal Science Research, Halal Products Research Institute, Universiti Putra Malaysia, Putra Inforport, Serdang 43400, Selangor, Malaysia or atikah1388@uitm.edu.my (N.A.H.A.N.); hafisyuswan@upm.edu.my (M.H.Y.); nadiahkarim@upm.edu.my (N.N.A.K.S.); 2 Faculty of Applied Sciences, Universiti Teknologi MARA, Cawangan Perlis, Kampus Arau, Arau 02600, Perlis, Malaysia; 3 Department of Process and Food Engineering, Faculty of Engineering, Universiti Putra Malaysia, Serdang 43400, Selangor, Malaysia; 4 Nutrition Unit, Institute for Medical Research, National Institutes of Health, No. 1, Jalan, Setia Murni U13/52, Seksyen U13 Setia Alam, Shah Alam 40170, Selangor, Malaysia; aswir@moh.gov.my; 5 Department of Formulation Design and Pharmaceutical Technology, Faculty of Pharmacy, Osaka Medical and Pharmaceutical University, 4-20-1 Nasahara, Takatsuki, Osaka 569-1094, Japan; kazunori.kadota@ompu.ac.jp

**Keywords:** *Caulerpa lentillifera*, color, D-optimal, hydrocolloid, jelly, k-carrageenan, locust bean gum, mixture design, pH, sugars

## Abstract

This study introduced a D-optimal design mixture to assess the physicochemical properties of a hydrocolloid-based functional food fortified with *C. lentillifera*. The combination incorporated vital jelly constituents, including extract (10–15%), sweeteners (20–29%), gelling agents (k-carrageenan and locust bean gum (LBG)), and preservatives (0–0.05%). The dependent variables were pH, Total Soluble Solid (TSS) value, and moisture content (MS). By employing the D-optimal design approach, a quadratic polynomial model was developed, demonstrating strong correlations with the experimental data with coefficient determinations (R^2^) of 0.9941, 0.9907, and 0.9989 for pH, TSS, and MS, respectively. Based on the D-optimal design, the study identified the optimum combination of significant factors with a desirability of 0.917, comprising 14.35% extract, 23.00% sucrose, 21.70% fructose, 26.00% k-carrageenan, 13.00% LBG, 1.95% CaCl_2_, and 0% methylparaben. The percentage of residual standard error (RSE) was less than 5%, indicating the reliability of the developed model. Furthermore, color analysis revealed significant differences among the jellies (*p* < 0.05). HPLC analysis demonstrated that the total sugar content in the fortified jellies was 28% lower compared to commercial jelly. Meanwhile, the bitterness level according to e-tongue showed a reduction of up to 90.5% when compared to the extract. These findings provide a valuable benchmark for the development of functional food products, ensuring their quality, safety, and extended shelf-life.

## 1. Introduction

Seaweed has been a part of the human diet for centuries: in Japan, it has been consumed since the fourth century, and in China, since the sixth century [1]. Today, seaweed aquaculture is a major industry in many Asian countries. In 2019, Asia produced 99% of the world’s seaweed, with China, Indonesia, and the Philippines being the leading producers [2]. Meanwhile, in the other part of the world such as Europe, although seaweed consumption is not as common as it is in Asia, it has gradually expanded in recent decades due to the discovery of its health benefits [3,4]. Of the more than 100 Caulerpa species that have been identified, only seven are edible. The most common edible Caulerpa species are *C. lentillifera* and *C. racemose* [1]. Some Caulerpa species, such as *C. cylindracea* and *C. toxifilia*, are invasive and may cause harm to the environment [5]. *C. racemosa* and *C. lentillifera*, also known as “sea grape” or “green caviar” [6], are usually eaten fresh as a snack, in salads and sushi, or in salt-preserved form in Japan, Korea, and Southeast Asian countries [7]. They are nutritious food sources and strong candidates to ensure food security for growing populations, especially in coastal tropical areas, due to their high nutritional composition, which includes polyunsaturated fatty acids, antioxidant activity, vitamins, minerals, and bioactive compounds. *C. lentillifera* is preferable as it is reproduced through fragmentation at lower costs of infrastructure or specialized expertise [8,9] and is better adapted in pond water compared to *C. racemosa* [10]. Similarly, to other seaweed species, *C. lentillifera* contains a lot of water, making exportation and transportation challenging because it could quickly deteriorate. Hence, the parental algae may encounter dehydration stress before reaching their destinations, increasing the likelihood of significant impacts. During storage and shipment, chlorophyll pigments might be totally or partially lost, where certain erect branches will turn white. In some cases, prolonged dehydration will lead to broken erect branches and the separation of spherical ramuli from erect branches [11]. Worst of all, *C. lentillifera* might lose its bioactive compounds and health benefits through the processes.

Thus, incorporating bioactive compounds into jellies as as a functional food has attracted scientists. Jellies are a ready-to-eat, shelf-stable food that can be transported almost anywhere, even into space, and are especially popular with kids [12]. Functional food based on jellies could also attract the elderly, for example, those with dysphagia who have difficulty swallowing pills and capsules [13,14]. Customers’ growing awareness of health and wellness goods, such as vitamins, minerals, and antioxidants, has included food containing bioactive substances [15]. The gel structures are essential, as they could give the food an appealing and distinctive texture and mouth feel [16].

In the development of jellies, hydrocolloids such as gelatin have always been used as gelling and foaming agents [17]. Gelatin is a product of the partial hydrolysis of collagen derived from animal skin, white connective tissue, and bones [18]. The primary sources of commercialized gelatin are cow or pig skins, which may be cheaper than other sources. However, this food is prohibited in some religions such as Islam, Judaism, and Hinduism. In addition, it is not suitable for vegetarians. Thus, driven by the demand for halal/kosher and gelatin-free products, the search has led to replacement with fish gelatin. In some cases, fish gelatin for example, is expensive and sometimes has a fishy odor. Substitution with plant hydrocolloids such as starch/modified starch, pectin, carrageenan, and agar has become an incredible alternative [18]. Of these choices, carrageenan has specific properties that differentiate it from other hydrocolloids, such as the ability to form gels with potassium and sodium ions, reactivity with milk protein, formation of thermoreversible gels, and synergism with different food hydrocolloids. In jellies, kappa carrageenan (k-carrageenan) is predominantly used in the Asian market. It is frequently combined with other gums, such as konjac or locust bean gum (LBG), to provide more mouthfeel and jelly texture experiences [19]. This clarifies why konjac or LBG are combined with carrageenan in jellies. 

Multivariate statistical analyses, such as response surface methodology (RSM) and Box–Behnken design (BBD), can be used to optimize factors that might influence formulation. However, D-optimal design is preferable, as it is the most suitable approach, especially for the food, pharmaceutical, and cosmeceutical industries. For example, it is suitable for identifying the desired characteristics and functional stability of the elements studied, as well as for statistically evaluating multiple variables and identifying interactions [20]. D-optimal design can reduce the number of experimental runs needed, which overcomes the disadvantages of traditional techniques. 

This study presents a valuable investigation into the physicochemical properties of a hydrocolloid-based functional food fortified with *C. lentillifera* using a D-optimal design mixture. It offers insights into the composition, structure, and behavior of the developed food, which can serve as a foundation for future research. The evaluation of key properties such as pH, total soluble solids, and moisture content improves quality control and food safety. Furthermore, the examination of color’s impact on the jellies’ composition enhances our understanding of consumer perception and taste expectations. By considering these vital elements, this study provides important information for the development of functional foods and advances the scientific community’s knowledge in this area.

## 2. Results and Discussion

### 2.1. Analysis of the Adequacy of the Fitted Model

In total, 17 runs of the D-optimal design were used to create the mixture of active components and excipients. The design maintains the excipients of interest and levels of the remaining formulation [21]. However, three runs in each response, specifically pH (8, 12), TSS (1, 6, 16), and MS (3, 6, 16), were marked as missing independent variables (outliers). These runs were not included in the construction of the model. Table 1 represents the number of runs and the responses obtained through the D-optimal design.

### 2.2. Agreement between Model Prediction and Observed Value

The agreement to fit in the models was evaluated through the Fisher test value (F-value), the *p*-value of the model, *p*-values of lack of fit, coefficient of determination (R^2^), adjusted coefficient of determination (Adjusted R^2^), predicted R^2^, and adequate precision of the model parameters and CV% from analysis of variance (ANOVA). Polynomial models were utilized to determine the ideal production conditions. Table 2 represents the parameter estimates and ANOVA of the model responses.

**Figure 1:**
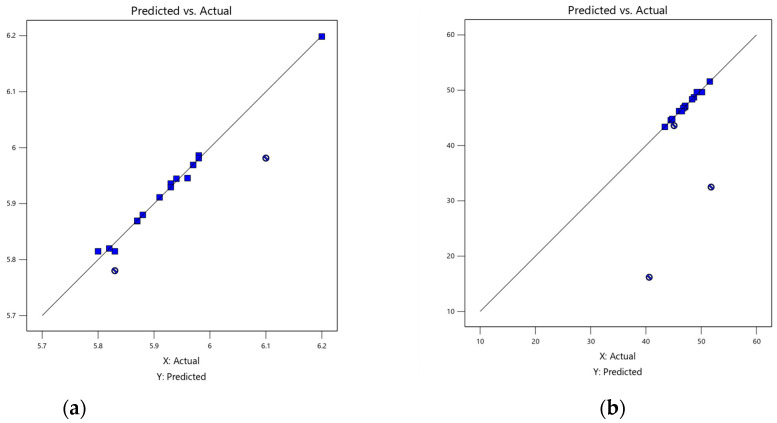

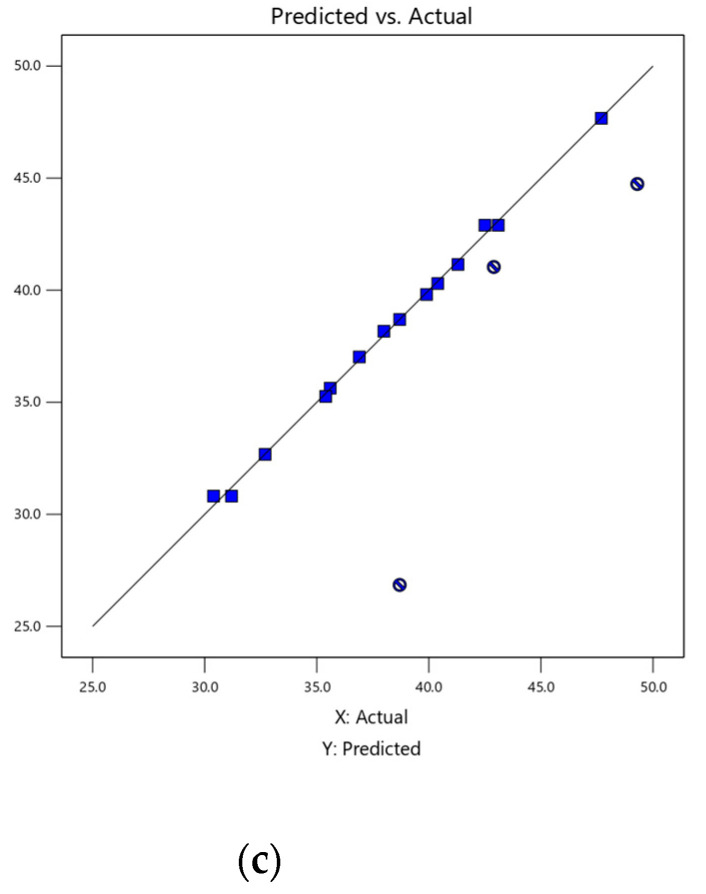
Predicted data versus actual values: (**a**) pH, (**b**) TSS, and (**c**) MS. The 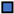 is the independent variables data, while 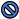 is missing independent variables.

The ANOVA results showed F-values of 94.31, 47.49, and 217.37 for the pH, TSS, and moisture content models were significant, respectively. There was only a 0.01% chance that an F-value this large could occur due to noise. This was supported by the finding that the probability is *p* < 0.05, indicating that the model was significant [22]. In addition, the lack of fit was not significant for all responses relative to the pure error. The high R^2^ of pH (0.9941), TSS (0.9907), and moisture content (0.9980) also demonstrated the presence of significant correlations. The high R^2^ readings indicated the quality of the fit for the selected models [23]. The significance of the adjusted R-square (Adj. R^2^) in assessing the model’s descriptive power was crucial. The values of Adj. R^2^ for pH, TSS, and MS were 0.9836, 0.9699, and 0.9934, respectively, indicating the predictability of the models in determining the optimum conditions necessary to achieve the desired outcomes for the jelly [22]. The findings verified the models’ predictability for determining the optimal conditions required to achieve the desired target, as shown in Figure 1.

The results showed that the adequate precision of all the responses was greater than 4, indicating that there was an adequate signal and that the model could be used to navigate the design space [24]. The coefficients of variation (CV) of the pH (0.21%), TSS (0.83%), and MS (1.04%) responses were acceptable, as they were less than 20%. The outcome demonstrated the reproducibility of the models.

The quadratic polynomial models for all responses are shown in Equations (1)–(3). The generated equations describe the empirical relationship between independent and dependent variables for each response. The positive values in the regression equation represent an effect that favors optimization due to the synergist. Negative values, on the other hand, indicate an antagonistic effect between the factors and responses [24]. In these equations, A represents the extract, B sucrose, C fructose, and G methylparaben. The linear coefficients of the pH model were all positive. However, the coefficients for TSS and MS were negative. This means that the interaction between sucrose and glucose could have a positive effect on the pH value of the jelly, but a negative effect on the TSS and MS values of the jelly. The coded equations for all responses are as follows:

pH = 6.01A + 5.87B + 5.88C + 5292.56G + 0.1291AB − 0.0297AC − 5237.68AG + 0.0973BC − 5324.94BG − 5334.20CG(1)

TSS = 36.48A + 51.58B + 48.72C − 1.139e + 06 G + 12.10AB + 8.07AC + 1.138e + 06AG − 10.27BC + 1.142 + 06BG + 1.145e + 06CG(2)

MS, % = 49.34A + 35.63B + 32.68C − 3.43e + 05G − 1.25AB − 1.31AC + 3.473e + 05AG − 21.65BC + 3.438e + 05BG + 3.467e + 05CG(3)

### 2.3. D-Optimal Analysis

The response surface design was established by D-optimal design using Design Expert Software, based on responses from the physicochemical analysis of the jellies. In the design, the range of ingredients used in the jellies was designated based on the values of the responses, and the response optimizer was used to meet the desired content of elements [25]. The reactions are also essential for adjusting the product and packaging and improving consumer acceptance in the future.

#### 2.3.1. pH Response Analysis

**Figure 2:**
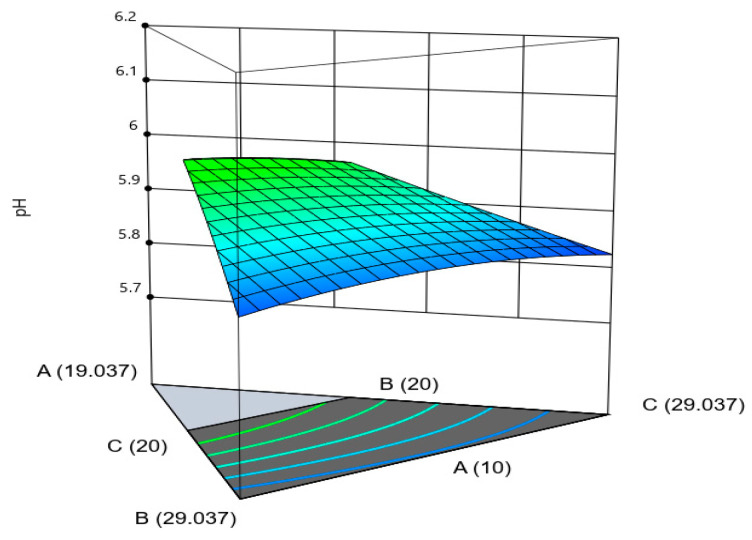
The three-dimensional surface shows the interaction between three variables with a significant impact on pH. (A): extract, (B): sucrose, (C): fructose.

In Figure 2, increasing the amount of extract and reducing the amount of sucrose produced the maximum pH values. The same antagonistic trend was observed in the interaction between the amounts of extract and fructose. The antagonistic effects between the amount of extract and sweeteners are likely caused by free hydrogen ion activity in the mixture [26]. A prior study also noted that pH decreased with additional sugar as a result of organic acid action [27]. However, an increasing amount of extract, sucrose, and fructose and a decreasing amount of methylparaben led to small changes in pH. This is likely because the range of methylparaben used was low.

The pH values of the fortified jellies in this study are higher than those of other jellies, which are typically between 2.7 and 3.6. This might be contributed to the pH of the extract. Similar pH in *Caulerpa* extract was also found to be alkaline, as reported [28]. A previous study found that higher pH values can reduce the rate of syneresis in jellies, while lower pH values can cause the molecules to lose bonding, resulting in a weak or runny gel [29]. 

However, too high a pH value could cause the gel to develop a liquid or syrup-like consistency. In addition, prolonged consumption of low-pH or acidic food (mostly below pH 4.9) can contribute to symptoms in the upper gastrointestinal tract such as ulcers and reflux. Acidic food can alter the gut bacteria’s viability due to unfavorable gastrointestinal physiological conditions (GI tract and bile secretions) [30]. To provide health benefits to the host, these bacteria must be viable upon consumption and have a substantial chance of surviving the journey to the GI tract [31]. The range of fortified jellies in this study has a suitable pH for the growth of lactic acid bacteria such as *Streptococcus thermophilus* (pH 6.5), *Lactobacillus bulgaris* (pH 5.8–6), and *Lactobacillus lactis subsp. cremoris* (pH 6.3–6.9) [32]. Based on the pH range, fortified jellies in this study have a strong potential to be prebiotic jellies and contribute to human health, similar to probiotics [33].

#### 2.3.2. Total Soluble Solid (TSS) Response Analysis

**Figure 3:**
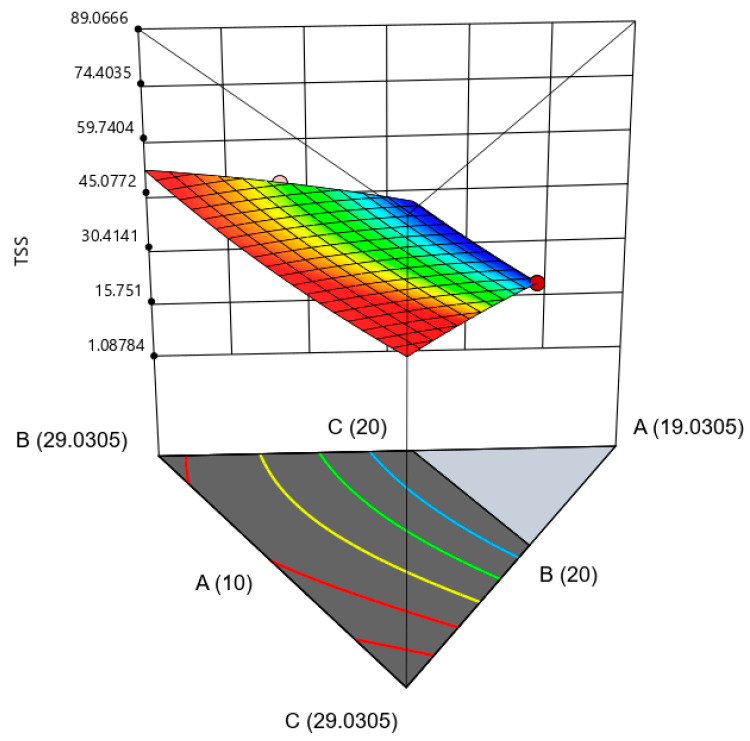
The three-dimensional surface shows the interaction between three variables with a significant impact on TSS. (A): extract, (B): sucrose, and (C): fructose.

The TSS values decreased in response to increasing amounts of extract and decreasing amounts of sucrose and fructose. This is expected, as the extract is a non-sugar component that could dilute the sugar concentration in the solution, reducing the TSS values. Furthermore, when sucrose and fructose are added to the mixture, they will completely dissolve in water and distribute the sugars evenly throughout the mixture. In contrast, extracts frequently contain ingredients or particles that are only partially soluble in water, which could cause them to settle to the bottom of the container. This may also lead to a lower TSS value due to a decreased sugar concentration in the solution. Figure 3 illustrates the three-dimensional surface shows the interaction between three variables on TSS.

Even though the TSS level in this study was slightly lower than that of earlier studies (60–65%), it was nonetheless equivalent to the TSS levels in commercially available jelly [34]. These variations could be due to the amount and type of sugar used in the mixture. These factors could influence the jelly structure rather than the TSS values. Too much sugar makes jelly solid and hard, whereas insufficient sugar weakens the jelly structure [35]. Likewise, high sugar consumption could contribute to the occurrence and development of fatty acid synthesis, dyslipidemia, insulin resistance, hyperuricemia, and cardiovascular diseases [36]. Therefore, it is preferable to incorporate a moderate amount of sugar in the mixture.

#### 2.3.3. Moisture Analysis

**Figure 4:**
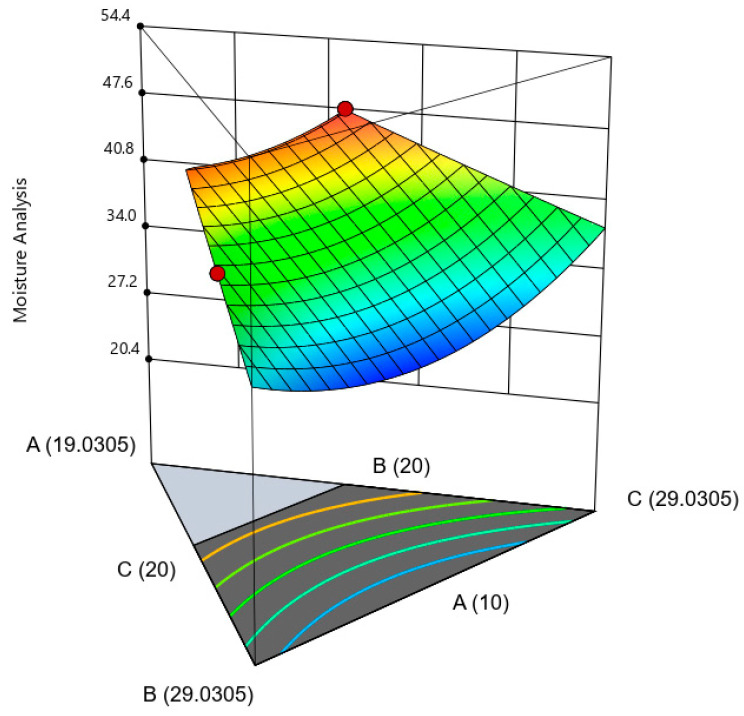
The three-dimensional surface shows the interaction between three variables with a significant impact on moisture content. (A): extract, (B): sucrose, and (C): fructose.

Moisture analysis is crucial as moisture is a “signal” that indicates the physical appearance in factors such as shape, color, texture, taste, weight, shelf life, freshness, quality, and resistance to bacterial contamination [37]. In Figure 4, the moisture content increased when high amounts of extract and low amounts of sucrose and fructose were used in the mixture. This is due to several reasons, including hygroscopicity, water activity, and the role of sugar as a humectant. The extracts are often hygroscopic, meaning they have a strong affinity for water molecules in the air. A high extract content in the mixture will attract moisture from the surroundings and contribute to the overall moisture content of the product. Meanwhile, the amount of water activity (aw) in the product could also increase the moisture content because the extract contains a significant amount of water. Low sugar levels may also increase overall moisture content due to the reduced availability of humectants to maintain the product’s moisture content. 

The moisture content of the fortified jellies was found to be comparable to the red dragon fruit peel jelly [38]. However, the moisture content of jellies can vary widely, with reported values being as low as (18%) [39] or as high as 93.83% [40]. The moisture content range for similar products could be due to several reasons, including the type and total amount of humectant, the types of sugars used, the processing conditions involved [37], the packaging and storage of the jellies, and the ingredients or raw materials used in the mixture. Adding gelling agents such as carrageenan to the mixture can indirectly affect the moisture content of the food by preventing the separation of liquid and solid elements in the food. The hydrocolloids from carrageenan can also reduce the amount of free water and bind a significant volume of water (H_2_O) due to their free OH ions, consequently strengthening bonding in the jellies [41]. A reduced amount of free water and moisture content within the food can significantly improve the shelf life of the food products. Carrageenan was also reported to be effective in capturing water during the gel-forming process compared to other hydrocolloids such as methoxyl pectin, carboxymethyl cellulose, and sago starch [42]. It is suggested to store food products in an environment with relative humidity (RH) of between 55% and 65% to prolong the shelf life of the food products [43].

### 2.4. Selection of the Design

The selected design for the mixture was predicted using Design Expert Software based on the results for the physicochemical properties of pH, TSS, and moisture content of the jellies. Desirability is defined as the precision of the mixture and the significance of each response [23]. In the selected mixture, the desirability value was 0.917, meaning that the combination used will produce a product with 91.7% of the characteristics of the desired target. This mixture consists of extract (14.35%), sucrose (23.00%), fructose (21.70%), k-carrageenan (26.00%), LBG (13.00%), CaCl_2_ (1.95%), and no methylparaben. With these proportions, the pH should be around 5.97, with TSS at 45.80% and moisture content at 40%.

### 2.5. Verification of Constructed Model

The experimental and predicted response values were compared by calculating the Residual Standard Error (RSE) percentages, as shown in Table 3. An RSE value of less than 5% indicates that the model is acceptable and the predicted and actual values are not significantly different.

### 2.6. Color Analysis

Color analysis is essential for jellies, as it influences the product’s aesthetic appeal and the perception of consumers. Thus, most food products in the market are often supplemented with artificial coloring, which generally offers more appealing than natural coloring [44]. Color analysis based on L*, a*, and b* of the jellies were compared using one-way analysis of variance (ANOVA), as shown in Supplementary Table S1.

There was a significant difference (*p* < 0.05) in the jellies’ L*, a*, and b*. Generally, the L* values of jellies with 15% of the extract are lower than those of jellies with 10% of the extract, which indicates that lightness decreased with increasing amounts of extract. The color from the extract of *C. lentillifera* may contribute to the color of the jelly. Caulerpa is rich in chlorophylls a and b and acts as a natural color additive in food and pharmaceutical products. In addition to adding value to the products, chlorophyll is an established antioxidant with demonstrated anti-cancer properties [45]. The natural color of *C. lentillifera* in the jellies could be a brilliant alternative, as prolonged consumption of synthetic dyes has been shown to be carcinogenic, result in hypersensitivity reactions, and cause behavioral issues, particularly in children [46].

In addition, the types and quantities of sweeteners and gelling agents used could also affect the results. A prior study found that the color values of carrageenan-based jellies can be affected by the type of sugar used, due to their molecular structure and properties [38]. For example, the sugar used can cause pigment destruction or non-enzymatic browning during heating. During thermal treatment, sucrose can degrade into glucose and fructose, which can promote the Maillard reaction. This reaction occurs among amino groups and reduces sugars under high temperatures, resulting in changes in the food’s aroma, taste, and color [47].

The chromatic coordinates a* and b* represent the color spaces between red (+a*) and green (−a*) and between yellow (+b*) and blue (−b*). The a* coordinates of the jellies ranged from −5.28 ± 1.28 to 0.40 ± 0.31. The extracted sample, however, has a positive a*. The low a* indicates that the jellies and extract are slightly green compared to red. The b* coordinates were positive in all the runs, indicating intense yellow coloration compared to blue. The same pattern of color coordination was observed in orange and berry gummies [48] when no artificial coloring was added.

### 2.7. Sugar Analysis Using HPLC

In addition, Total Soluble Solids (TSS), and the details of monosaccharides and saccharides in the jelly were also obtained. The jellies were chosen based on the high, medium, and low amounts of extract used in the mixture. Table 4 shows the types of monomers and their quantities present.

The total sugar content of the fortified jellies was significantly different (*p* < 0.05) from the control jelly (without the extract) and the extract of *C. lentillifera*. The total sugar content of the fortified jellies was reduced by up to 28%, compared to the control jelly (without the extract) and commercial market products. This provides a better dietary option that can reduce the risk of health problems such as obesity, metabolic dysregulation, cardiovascular disease, and type 2 diabetes [49].

This study found that the fortified jellies had a higher glucose content than the extract alone. The increase in glucose content in the jellies may be attributed to the sucrose and fructose in the mixture. However, the glucose content in the fortified jellies was lower than in commercial products, which had a glucose content of 11.937 ± 0.00%. Glucose is an essential nutrient that maintains glucose homeostasis, which is vital to the normal physiology of cells [50]. A previous study reported that k-carrageenan can reduce blood glucose in diabetic male Wistar rats (*Ratus norvegicus*) [51]. Glucose is an important energy source that is the primary fuel for the brain and muscles. It can regulate blood sugar levels, which is beneficial for people with diabetes or at risk of developing diabetes. Glucose can also improve gut health by acting as a prebiotic to aid in digestion and boost immunity by supporting the production of white blood cells. However, it is important to consume glucose in moderation, as excessive amounts can lead to obesity and other health problems.

The fructose content was reduced in all fortified jellies, while the sucrose content was increased due to the addition of sucrose as one of the ingredients in the mixture. The sweetness of the jellies was achieved by the glycosidic bond between glucose and fructose, which is formed between C1 and C2 of glucosyl and fructosyl in the sucrose molecule [52]. The sugars in jelly bind directly to k-carrageenan to stabilize the intermolecular hydrogen bonding between individual strands in a typical junction zone. This is achieved by forming intermolecular, cross-linking hydrogen bonds between the OH groups of the sugar cosolvent and k-carrageenan [53,54]. Sucrose has been found to have a positive effect on the quality and stability of gels. Previous studies have elucidated that adding sucrose can increase the gelation temperature of mixtures and improve the freeze–thaw stability and combination of tapioca starch [55]. Additionally, sucrose has been found to improve the strength and thermal stability of azuki bean starch (ABS) gels [56].

### 2.8. E-Tongue

**Figure 5:**
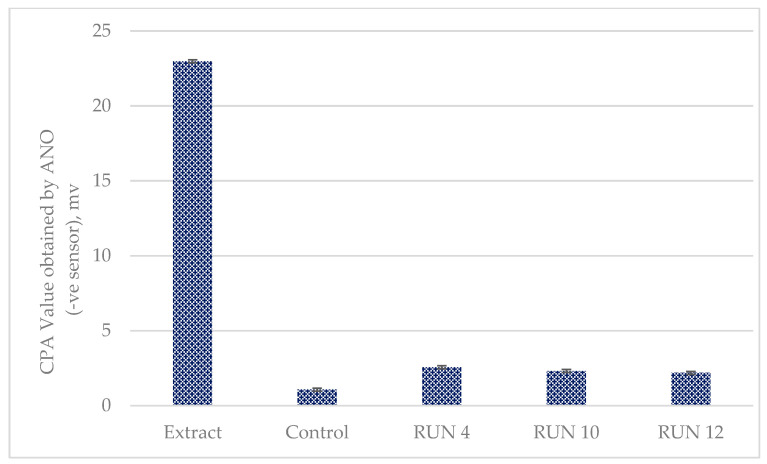
Comparison of the change in membrane potential caused by adsorption (CPA) values obtained using an ANO sensor on selected runs. Each bar represents the mean ± standard error of the triplicate tests.

Electronic tongues are a reliable instrument for assessing the bitterness of medicines in the pharmaceutical sector [56,57]. Figure 5 compares the change in membrane potential caused by adsorption (CPA) values obtained using an ANO sensor.

The bitterness levels of the extract and the fortified jellies were compared based on the CPA values measured with the ANO sensor. CPA stands for change in membrane potential caused by adsorption, which corresponds to the aftertaste. The ANO sensor was composed of a lipid-phosphoric acid didodecyl ester of lipid and a plasticizer-dioctylphenyl phosphate [58,59]. Therefore, the ANO sensor is sensitive to basic materials such as solifenacin succinate or amplopodone besilate [60]. This sensor can also detect various bitter substances and taste sensors that detect non-charged bitter substances. The results for the extract and the fortified jellies in this study showed a significant difference (*p* < 0.05). However, there was no significant difference (*p* > 0.05) between Runs 4, 10, and 12. Although the fortified jellies have a slightly basic pH, it was possible to successfully reduce the bitterness intensity level of *C. lentillifera* from 22.97 mv to 2.56 mv (Run 4), 2.31 mv (Run 10), and 2.18 mv (Run 12), which represent approximate reductions of 88.85%, 89.94%, and 90.50%, respectively. This is due to the sugars [61] and gelling agents [62] used in the mixture, which masked the bitterness of the extract. This is consistent with a previous study, which showed that sugars can decrease bitterness and intensify sweetness in food [61] and conceal the bitter taste in active pharmaceutical ingredients [63]. Sugars can counteract bitter flavors by activating the taste receptors on the tongue that are responsible for detecting sweetness [64].

Previous research has shown that gelling agents such as alginate beads can effectively mask the bitter taste of *Momordica charantia* extract [62]. Other gelling agents, such as xantham gum, locust bean gum, and agar have been reported to reduce the bitterness of ambroxol hydrochloride [65]. The gelling agents will modify the overall texture of the jelly, masking the unpleasant taste of the extract and creating a smoother and more cohesive mouthfeel. This can help to improve overall palatability [65].

## 3. Conclusions

A D-optimal design mixture was successfully applied to develop a hydrocolloid-based functional food fortified with *C. lentillifera* and evaluate its physicochemical properties. The ANOVA and R^2^ values of the pH, TSS, and MS were 0.9941, 0.9907, and 0.9989, respectively. This indicates that the developed quadratic models were well-formed. The desirability values of 0.917 suggest that actual values under the optimum conditions (14.35% extract, 23.00% sucrose, 21.70% fructose, 26.00% k-carrageenan, 13.00% LBG, 1.95% CaCl_2_, and without methylparaben) corresponded closely to the predicted values, as the RSE percentage was less than 5%. Furthermore, significant (*p* < 0.05) color variations were found among the jellies. The total amount of sugar in the fortified jellies was up tp to 28% lower than commercial jelly. This gives consumers the opportunity to make healthier choices and avoid health problems, such as obesity and its associated consequences. Additionally, the pH range of the jellies in this study suggests that they have strong potential as prebiotic jellies, which can have a positive impact on human health. Through e-tongue analysis, the fortified jellies were able to mask the bitterness level of *C. lentillifera* by up to 90.5%.

## 4. Materials and Methods

### 4.1. Experimental Materials

*Caulerpa lentillifera* was harvested in Port Dickson, Negeri Sembilan, Malaysia. Sucrose and fructose were purchased from a local bakery, while k-carrageenan, CaCl_2,_ and locust bean gum (LBG) were procured from Modernist Pantry, LLC (Eliot, ME, USA). All ingredients are food grade.

### 4.2. Extraction of C. lentillifera

Water extraction was performed by Ultrasonic Assisted Extraction (UAE) using a Branson Digital Sonifier (Danbury, CT, USA), with a sample-to-solvent ratio of 1:20. The parameters were set at 40% amplitude for 7 min. The extracts were filtered using a Whatman filter paper (No. 1) and maintained at −20 °C until needed.

### 4.3. Selection of Excipients

The pH of the jellies is the vital element that needs to be considered because jellies will not solidify under low pH conditions. In addition, oral jelly must have significant viscosity and a soft structure to be easily squeezed out of the sachets [62]. Therefore, structure, sweeteners, and product shelf life must be considered as they will affect the customer’s preferences. Sucrose and fructose were used in the mixture because they are cost-effective, have a mild flavor, and prevent crystallization [65]. Sugar is a structural agent that increases viscosity, helps gels to form, and keeps them firm. Other sugar alternatives might not have the same properties, and additional structures may be required. In addition, fructose is the most soluble monosaccharide sugar, so it is less likely to crystallize, which is why it is often used in high-sugar foods and beverages to preserve their desirable textures [66]. In addition, non-nutritive sugars such as saccharin, sucralose, and aspartame have been shown to have negative effects on urinary bladder tumors and gut microbiota [48]. k-carrageenan has the best solidifying capability and is also thermo-reversible, anti-protein coagulating, hydrophilic, non-toxic, biodegradable, and low-cost compared to other types of carrageenan and has garnered much interest in the food, chemical, packaging, pharmaceutical, and edible cling film industries. This is due to the 3,6-anhydrous-D-galactopyranose residues in the chain and one negatively charged sulfate group [67]. To improve the gel strength, texture, and syneresis the k-carrageenan and locust bean gum (LBG) were added to the mixture. The combination of these gelling and thickening agents is known to work synergistically [68]

### 4.4. Preparation of Jellies Fortified with C. lentillifera

The fortified jellies were prepared via heating and congealing methods, as reported [66]. The amounts of extract, gelling agents, and stabilizers were measured according to Table 1. Thickening and emulsifying agents k-carrageenan and locust bean gum was prepared separately at a ratio of 2:1 before being added to the mixture of sucrose and fructose. When the mixture was dissolved completely, a stabilizer, methylparaben, and extract were added and stirred again to enhance the softness of the jelly for a few minutes. The mixture was transferred into molds that were 7.3 cm × 1.5 cm × 1.5 cm and allowed to solidify for 24 h at 4 °C, covered with plastic wrap to avoid exposure to the outer environment.

### 4.5. Physicochemical Analysis

The pH of the fortified jellies was measured using a Mettler Toledo (Columbus, OH, USA) desk pH meter. Total Soluble Sugar (TSS) was measured in terms of refractive index and concentration (% Brix) of the mixture, using a digital refractometer (Hanna Instrument, HI 96800, Cincinnati, OH, USA). Moisture content was measured using an Infrared Moisture Analyser FD-660 (Kett Electric Laboratory Co. Ltd., Tokyo, Japan), and color analysis was conducted using a WR-10 portable handheld color meter (FRU, Guangzhou, China), based on lightness (L*), and chromatic values (a* and b*). All measurements were conducted in triplicate.

### 4.6. Sugar Analysis

High-Performance Liquid Chromatography (HPLC) was performed using 5% of standard solutions (fructose, glucose, sucrose, and maltose) dissolved in 50:50 acetonitrile/water (ACN/dH_2_O). The working sugar mixture solution was prepared by transferring 1 mL of each standard solution to a 10 mL volumetric flask, and then the final volume was completed with distilled water. Meanwhile, the fortified jelly was weighed to 1 g, dissolved in 25 mL of 50:50 (ACN/dH_2_O) and homogenized. The samples were then centrifuged for 30 min at 3200 rpm before being filtered using a 0.45 µm nylon syringe filter. About 1 mL of the sample was then transferred to the HPLC vial. Compounds were separated using a Cosmosil ^®^ 5C18-PAQ Packed Column 4.6 mml. D. × 250 mm, 1PKG (Nacalai Tesque, Kyoto, Japan). The mobile phase consisted of (ACN/dH_2_O) (50:50 *v*/*v*) in isocratic mode at a 1.0 mL/min flow rate and injection volume of 10 μL.

### 4.7. Electric Tongue Sensor

SA402B (Intelligent Sensor Technology, Inc., Kanagawa, Japan), was used to test the bitterness intensity of the fortified jellies at the Formulation Design and Pharmaceutical Technology Laboratory, Faculty of Pharmacy, Osaka Medical and Pharmaceutical University (OMPU). The ANO sensor, which responds to the specific bitterness of hydrochloride, was used to determine the bitterness of the jellies. The negatively charged membrane of the ANO sensor makes it extremely sensitive to hydrochloride [57,67].

### 4.8. Experimental Design and Statistical Analysis

The pH, TSS, and MS of the fortified jellies were evaluated using Design Expert software (Version 13.0.1, Stat-Ease, Inc. Minneapolis, MN, USA), while the color, sugar, and bitterness levels of the jellies were analyzed through Minitab Statistical software. Optimization was carried out to obtain an optimal response according to the desired optimization target. The essential parameters were determined so that a solution formula could be identified during optimization to produce a solution mixture that would be chosen based on the highest degree of desirability, which ranges from 0 to 1 [23]. Table 5 lists the excipient proportion limitations in the fortified jelly.

### 4.9. Verification of the Model

A quantitative comparison between the obtained experimental and theoretical prediction values was performed to validate the models through residual standard error (RSE). At the same time, the percentage of the calculated values was determined. The predicted error was calculated according to the differences between the experimental values and the predicted value per predicted value [21].

## Figures and Tables

**Table 1:** The mixtures and responses of the fortified jellies from the D-optimal mixture design.

No.	A	B	C	D	E	F	G	pH	TSS, %	MS, %
1	12.50	24.89	21.63	26	13	1.95	0.03	5.94	45.1	38.7
2	15.00	22.03	22.02	26	13	1.95	0	5.97	41.5	41.3
3	13.75	21.63	23.66	26	13	1.95	0.01	5.96	48.0	42.9
4	12.50	23.28	23.27	26	13	1.95	0	5.93	47.4	35.4
5	12.50	20.00	26.55	26	13	1.95	0	5.91	48.5	36.9
6	15.00	24.00	20.00	26	13	1.95	0.05	6.20	43.1	49.3
7	12.50	26.53	20.00	26	13	1.95	0.02	5.93	45.6	40.4
8	10.00	20.00	29.00	26	13	1.95	0.05	5.83	48.8	39.9
9	10.00	29.05	20.00	26	13	1.95	0	5.87	50.1	35.6
10	10.00	24.51	24.51	26	13	1.95	0.03	5.83	49.7	30.4
11	15.00	24.05	20.00	26	13	1.95	0	6.10	45.0	42.5
12	15.00	24.05	20.00	26	13	1.95	0	5.98	44.5	43.1
13	10.00	20.00	29.05	26	13	1.95	0	5.88	48.7	32.7
14	15.00	20.00	24.03	26	13	1.95	0.02	5.98	44.5	47.7
15	10.00	29.00	20.00	26	13	1.95	0.05	5.82	51.8	38.7
16	10.00	29.00	20.00	26	13	1.95	0.05	5.84	52.5	39.2
17	11.25	21.63	26.13	26	13	1.95	0.04	5.87	45.5	38.0

Extract (A), sucrose (B), fructose (C), carrageenan (D), locust bean gum (E), CaCl_2_ (F), and methylparaben (G).

**Table 2:** Analysis of variance (ANOVA) and *p*-values of the physical analysis of the fortified jellies.

Sources	Physical Parameters		
	pH	TSS, %	MS, %
Model	Significant	Significant	Significant
R^2^	0.9941	0.9907	0.9980
Adjusted R^2^	0.9836	0.9699	0.9934
Predicted R^2^	0.9354	0.9344	0.9226
Adequate Precision	38.2014	25.0094	50.4348
*p*-value	<0.0001	0.0010	<0.0001
*F* value	94.31	47.49	217.37
C.V. %	0.21	0.83	1.04
Standard deviation	0.0123	0.3880	0.3955
PRESS	0.0083	4.26	0.9980

**Table 3:** The actual and predicted data for the model verification.

Independent Variables								Responses								
								pH			TSS			MS		
No.	A	B	C	D	E	F	G	X	Y	RSE	X	Y	RSE	X	Y	RSE
1	14.3	23.0	21.7	26.0	13.0	1.9	0	5.97	5.96	0.17	45.8	44.8	2.18	40.0	38.4	4.00
2	14.1	23.0	21.8	26.0	13.0	1.9	0	5.96	5.93	0.50	45.9	46.8	1.96	39.5	37.8	4.30
3	14.1	23.5	21.3	26.0	13.0	1.9	0	5.97	5.94	0.51	46.3	46.8	1.07	39.9	38.2	4.26

Note: (A): extract, (B): sucrose, (C): fructose, (D): k-carrageenan, (E): LBG, (F): CaCl_2_ (G): methylparaben, (X): predicted value, (Y): actual value, and RSE (%).

**Table 4:** Sugar analysis of jelly using HPLC.

Sample/Sugar	Fructose	Glucose	Sucrose	Maltose	Total, %
Jelly Run 4	0.22 ± 0.01	4.19 ± 0.054	25.17 ± 0.48	2.77 ± 0.09	32.37
Jelly Run 10	0.18 ± 0.00	4.52 ± 0.062	26.91 ± 0.42	3.01 ± 0.72	34.63
Jelly Run 12	0.20 ± 0.035	3.89 ± 0.074	24.35 ± 1.21	2.55 ± 0.06	31.00
Control	ND	4.72 ± 0.035	29.76 ± 0.473	3.22 ± 0.13	37.73
Extract	3.687 ± 0.04	3.754 ± 0.023	ND	ND	7.37
Commercial jelly	1.23 ± 0.00	11.937 ± 0.00	24.658 ± 0.07	5.70 ± 0.16	43.53
*p*-value	0.0000	0.0000	0.0000	0.0000	

ND: not detected.

**Table 5:** Summary of excipient proportion limitations.

Causal Factor Variables	Coded Level of Variable (%)	
	Low (−1)	High (+1)
Extract	10	15
Sucrose	20	29.05
Fructose	20	29.05
Methylparaben	0	0.05

The total amount in the mixture is in percentages: (A) extract + (B) sucrose + (C) fructose + (D) (methylparaben) + other ingredients = 100%.

## Data Availability

Data sharing is not applicable.

## Supplementary Materials

The following supporting information can be downloaded at: https://www.mdpi.com/article/10.3390/gels9070531/s1, Table S1: The color analysis of the fortified jellies.

## References

[1] Syakilla N., George R., Chye F.Y., Pindi W., Mantihal S., Wahab N.A., Fadzwi F.M., Gu P.H., Matanjun P. (2022). A Review on Nutrients, Phytochemicals, and Health Benefits of Green Seaweed, *Caulerpa lentillifera*. Foods.

[2] Biancacci C., Visch W., Callahan D.L., Farrington G., Francis D.S., Lamb P., McVilly A., Nardelli A., Sanderson J.C., Schwoerbel J. (2022). Optimisation of At-Sea Culture and Harvest Conditions for Cultivated Macrocystis Pyrifera: Yield, Biofouling and Biochemical Composition of Cultured Biomass. Front. Mar. Sci..

[3] Mabeau S., Fleurence J. (1993). Seaweed in Food Products: Biochemical and Nutritional Aspects. Trends Food Sci. Technol..

[4] Sanjeewa K.K.A., Lee W.W., Jeon Y.J. (2018). Nutrients and Bioactive Potentials of Edible Green and Red Seaweed in Korea. Fish. Aquat. Sci..

[5] du Preez R., Majzoub M.E., Thomas T., Panchal S.K., Brown L. (2020). *Caulerpa lentillifera* (Sea Grapes) Improves Cardiovascular and Metabolic Health of Rats with Diet-Induced Metabolic Syndrome. Metabolites.

[6] Yap W.F., Tay V., Tan S.H., Yow Y.Y., Chew J. (2019). Decoding Antioxidant and Antibacterial Potentials of Malaysian Green Seaweeds: *Caulerpa racemosa* and *Caulerpa lentillifera*. Antibiotics.

[7] Long H., Gu X., Zhu Z., Wang C., Xia X., Zhou N., Liu X., Zhao M. (2020). Effects of Bottom Sediment on the Accumulation of Nutrients in the Edible Green Seaweed *Caulerpa lentillifera* (Sea Grapes). J. Appl. Phycol..

[8] de Gaillande C., Payri C., Remoissenet G., Zubia M. (2017). Caulerpa Consumption, Nutritional Value and Farming in the Indo-Pacific Region. J. Appl. Phycol..

[9] Stuthmann L.E., Springer K., Kunzmann A. (2021). Cultured and Packed Sea Grapes (*Caulerpa lentillifera*): Effect of Different Irradiances on Photosynthesis. J. Appl. Phycol..

[10] Tanduyan S.N., Gonzaga R.B., Bensig V.D. (2013). Off Bottom Culture of *Caulerpa lentillifera* in Three Different Water Levels in the Marine Waters of San Francisco, Cebu, Philippines. Galaxea J. Coral Reef Stud..

[11] Liang Z., Liu F., Wang W., Zhang P., Yuan Y., Yao H., Sun X., Wang F. (2021). A Reasonable Strategy for *Caulerpa lentillifera* J. Agardh (Bryopsidales, Chlorophyta) Transportation Based on the Biochemical and Photophysiological Responses to Dehydration Stress. Algal Res..

[12] Shinwari K.J., Rao P.S. (2018). Stability of Bioactive Compounds in Fruit Jam and Jelly during Processing and Storage: A Review. Trends Food Sci. Technol..

[13] Crary M., Sura L., Madhavan A., Carnaby-Mann G. (2012). Dysphagia in the Elderly: Management and Nutritional Considerations. Clin. Interv. Flanging.

[14] Fujioka S., Kadota K., Yoshida M., Shirakawa Y. (2020). Improvement in the Elution Behavior of Rutin via Binary Amorphous Solid with Flavonoid Using a Mechanochemical Process. Food Bioprod. Process..

[15] Miranda J.S., Costa B.V., de Oliveira I.V., de Lima D.C.N., Martins E.M.F., de Castro Leite Júnior B.R., do Nascimento Benevenuto W.C.A., de Queiroz I.C., da Silva R.R., Martins M.L. (2020). Probiotic Jelly Candies Enriched with Native Atlantic Forest Fruits and Bacillus Coagulans GBI-30 6086. Lwt.

[16] Shahsavani Mojarrad L., Rafe A. (2018). Rheological Characteristics of Binary Composite Gels of Wheat Flour and High Amylose Corn Starch. J. Texture Stud..

[17] Nogami S., Uchiyama H., Kadota K., Tozuka Y. (2021). Design of a PH-Responsive Oral Gel Formulation Based on the Matrix Systems of Gelatin/Hydroxypropyl Methylcellulose Phthalate for Controlled Drug Release. Int. J. Pharm..

[18] Karim A.A., Bhat R. (2008). Gelatin Alternatives for the Food Industry: Recent Developments, Challenges and Prospects. Trends Food Sci. Technol..

[19] Wang X., Zhou D., Guo Q., Liu C. (2021). Textural and Structural Properties of a *κ*-carrageenan–Konjac Gum Mixed Gel: Effects of *κ*-carrageenan Concentration, Mixing Ratio, Sucrose and Ca^2+^ Concentrations and Its Application in Milk Pudding. J. Sci. Food Agric..

[20] Borhan F.P., Abd Gani S.S., Shamsuddin R. (2014). The Use of D-Optimal Mixture Design in Optimising Okara Soap Formulation for Stratum Corneum Application. Sci. World J..

[21] Jesus R.R.-A., Ariadna L.P., Julio C. (2015). sar E. A.; Humberto, M.Q.; Antonio, I.C.; Jose, C.T.C. Optimization of a Novel Tablets Formulation Using D-Optimal Mixture Design. Afr. J. Pharm. Pharmacol..

[22] Zakaria F., Tan J.K., Mohd Faudzi S.M., Abdul Rahman M.B., Ashari S.E. (2021). Ultrasound-Assisted Extraction Conditions Optimisation Using Response Surface Methodology from Mitragyna Speciosa (Korth.) Havil Leaves. Ultrason. Sonochem..

[23] Sriharti, Andriansyah R.C.E., Agustina W., Indriati A., Litaay C., Luthfiyanti R., Mayasti N.K.I., Triyono A., Tribowo R.I., Purwandoko P.B. (2022). Optimization of Herbal Tea Drink Formula Based on Aloe Vera Rind (*Aloe barbadensis miller*). Food Sci. Technol..

[24] Kamairudin N., Gani S., Masoumi H., Hashim P. (2014). Optimization of Natural Lipstick Formulation Based on Pitaya (*Hylocereus polyrhizus*) Seed Oil Using D-Optimal Mixture Experimental Design. Molecules.

[25] Rasidek N.A.M., Nordin M.F.M., Shameli K. (2016). Formulation and Evaluation of Semisolid Jelly Produced by *Musa acuminata* Colla (AAA Group) Peels. Asian Pac. J. Trop. Biomed..

[26] Schiassi M.C.E.V., Salgado D.L., Meirelles B.S., Lago A.M.T., Queiroz F., Curi P.N., Pio R., de Souza V.R. (2019). Berry Jelly: Optimization Through Desirability-Based Mixture Design. J. Food Sci..

[27] Lim J.-H., Park S.-S., Jeong J.-W., Park K.-J., Seo K.-H., Sung J.-M. (2013). Quality Characteristics of Kimchi Fermented with Abalone or Sea Tangle Extracts. J. Korean Soc. Food Sci. Nutr..

[28] Harwanto D., Saputro P., Susilowati T., Haditomo A.H.C., Windarto S. (2020). Effect of Different n:P Ratios Application on the Cultivation Media for the Growth and Fiber Content of *Caulerpa racemosa* Reared in Tarpaulin Ponds. AACL Bioflux.

[29] Everard C.D., O’Callaghan D.J., Fagan C.C., O’Donnell C.P., Castillo M., Payne F.A. (2007). Computer Vision and Color Measurement Techniques for Inline Monitoring of Cheese Curd Syneresis. J. Dairy Sci..

[30] Schultrich K., Henderson C.J., Braeuning A., Buhrke T. (2020). Correlation between 3-MCPD-Induced Organ Toxicity and Oxidative Stress Response in Male Mice. Food Chem. Toxicol..

[31] Sahadeva R.P.K., Leong S.F., Chua K.H., Tan C.H., Chan H.Y., Tong E.V., Wong S.Y.W., Chan H.K. (2011). Survival of Commercial Probiotic Strains to PH and Bile. Int. Food Res. J..

[32] Rault A., Bouix M., Béal C. (2009). Fermentation PH Influences the Physiological-State Dynamics of *Lactobacillus bulgaricus* CFL1 during PH-Controlled Culture. Appl. Environ. Microbiol..

[33] Pasolli E., De Filippis F., Mauriello I.E., Cumbo F., Leech J., Cotter P.D., Segata N., Ercolini D., Walsh A.M. (2020). Bacteria from Food With the Gut Microbiome. Nat. Commun..

[34] Ventura E.E., Davis J.N., Goran M.I. (2011). Sugar Content of Popular Sweetened Beverages Based on Objective Laboratory Analysis: Focus on Fructose Content. Obesity.

[35] Featherstone S. (2016). Jams, Jellies, and Related Products. A Complete Course in Canning and Related Processes.

[36] Stanhope K.L. (2016). Sugar Consumption, Metabolic Disease and Obesity: The State of the Controversy. Crit. Rev. Clin. Lab. Sci..

[37] Vera M., Dutta B., Mercer D.G., Maclean H.L., Touchie M.F. (2019). Assessment of Moisture Content Measurement Methods of Dried Food Products in Small-Scale Operations in Developing Countries: A Review. Trends Food Sci. Technol..

[38] Soedirga L.C., Marchellin M. (2021). Physicochemical Properties of Jelly Candy Made with Pectin from Red Dragon Fruit Peel in Combination with Carrageenan. Caraka Tani J. Sustain. Agric..

[39] Faridah A. (2019). The Effect of Water Extract of Brown Seaweed on the Characteristic of Jelly Candy as a Functional Food. Int. J. Res. Rev..

[40] Hwang E.-S., Moon S.J. (2021). Quality Characteristics and Antioxidant Activity of Stick Jelly Made with Different Amount of Tomato Juice. J. Korean Soc. Food Sci. Nutr..

[41] Setiaboma W., Fitriani V., Mareta D.T. (2019). Characterization of Fruit Leather with Carrageenan Addition with Various Bananas. IOP Conf. Ser. Earth Environ. Sci..

[42] Amin P., Riyadi P.H., Kurniasih R.A., Husni A. (2022). Utilization of κ-Carrageenan as Stabilizer and Thickener of Honey Pineapple (Ananas Comosus [L. Merr]) Jam. Food Res..

[43] Yusof N., Jaswir I., Jamal P., Jami M.S. (2019). Texture Profile Analysis (TPA) of the Jelly Dessert Prepared from Halal Gelatin Extracted Using High Pressure Processing (HPP). Malays. J. Fundam. Appl. Sci..

[44] Giger A. (2002). Chemical Synthesis Project. A New Yellow Carotenoid. Pure Appl. Chem..

[45] Paul N.A., Neveux N., Magnusson M., de Nys R. (2014). Comparative Production and Nutritional Value of “Sea Grapes”—The Tropical Green Seaweeds *Caulerpa lentillifera* and *C. racemosa*. J. Appl. Phycol..

[46] Potera C. (2010). Forum the Artificial Food Dye Blues Invasion of the Bedbugs. Environ. Health Perspect..

[47] Smejkal Q., Fiedler T., Kurz T., Kroh L. (2007). Simplified Kinetics and Colour Formation in Sucrose Solutions Based on A-Dicarbonyl Compounds. Int. J. Food Eng..

[48] Teixeira-Lemos E., Almeida A.R., Vouga B., Morais C., Correia I., Pereira P., Guiné R.P.F. (2021). Development and Characterization of Healthy Gummy Jellies Containing Natural Fruits. Open Agric..

[49] Satokari R. (2020). High Intake of Sugar and the Balance between Pro- and Anti-Inflammatory Gut Bacteria. Nutrients.

[50] Chen C., Lu Y., Yu H., Chen Z., Tian H. (2019). Influence of 4 Lactic Acid Bacteria on the Flavor Profile of Fermented Apple Juice Influence of 4 Lactic Acid Bacteria. Food Biosci..

[51] Surini S., Diandra D.M. (2017). Formulation of Mulberry Leaf (*Morus Alba* L.) Extract Hydrogel Beads Using Cross-Linked Pectin. Int. J. Appl. Pharm..

[52] Alasalvar C., Pelvan E., Özdemir K.S., Kocadağlı T., Mogol B.A., Paslı A.A., Özcan N., Özçelik B., Gökmen V. (2013). Compositional, Nutritional, and Functional Characteristics of Instant Teas Produced from Low- and High-Quality Black Teas. J. Agric. Food Chem..

[53] Stenner R., Matubayasi N., Shimizu S. (2016). Gelation of Carrageenan: Effects of Sugars and Polyols. Food Hydrocoll..

[54] Watase M., Nishinari K. (1985). Differential Scanning Calorimetry and Large Deformation Behaviour of Kappa-Carrageenan Gels Containing Alkali Metal Ions. Colloid Polym. Sci..

[55] Tunnarut D., Pongsawatmanit R. (2017). Quality Enhancement of Tapioca Starch Gel Using Sucrose and Xanthan Gum. Int. J. Food Eng..

[56] Choi D.H., Kim N.A., Nam T.S., Lee S., Jeong S.H. (2014). Evaluation of Taste-Masking Effects of Pharmaceutical Sweeteners with an Electronic Tongue System. Drug Dev. Ind. Pharm..

[57] Kadota K., Nogami S., Uchiyama H., Tozuka Y. (2020). Controlled Release Behavior of Curcumin from Kappa-Carrageenan Gels with Flexible Texture by the Addition of Metal Chlorides. Food Hydrocoll..

[58] Harada T., Uchida T., Yoshida M., Kobayashi Y., Narazaki R., Ohwaki T. (2010). A New Method for Evaluating the Bitterness of Medicines in Development Using a Taste Sensor and a Disintegration Testing Apparatus. Chem. Pharm. Bull..

[59] Ito M., Ikehama K., Yoshida K., Haraguchi T., Yoshida M., Wada K., Uchida T. (2013). Bitterness Prediction of H1-Antihistamines and Prediction of Masking Effects of Artificial Sweeteners Using an Electronic Tongue. Int. J. Pharm..

[60] Uno R., Ohkawa K., Kojima H., Haraguchi T., Ozeki M., Kawasaki I., Yoshida M., Habara M., Ikezaki H., Uchida T. (2023). Masking the Taste of Fixed-Dose Combination Drugs: Particular NSAIDs Can Efficiently Mask the Bitterness of Famotidine. Chem. Pharm. Bull..

[61] Takagi A., Kubo R., Jibiki A., Aomori T., Suzuki S., Nakamura T. (2017). Identification of Foods Masking Clindamycin Bitterness Using an Electronic Gustatory Screening System Followed by Organoleptic Examination. Iryo Yakugaku (Jpn. J. Pharm. Health Care Sci.).

[62] Sutriyo S., Iswandana R., Fauzi F. (2018). Strategy to Mask the Bitter Taste of Momordica Charantia Extract Using Alginate–Gelatin Beads. Int. J. Appl. Pharm..

[63] Bin L.K., Gaurav A., Mandal U.K. (2019). A Review on Co-Processed Excipients: Current and Future Trend of Excipient Technology. Int. J. Pharm. Pharm. Sci..

[64] Kuhn C., Bufe B., Winnig M., Hofmann T., Frank O., Behrens M., Lewtschenko T., Slack J.P., Ward C.D., Meyerhof W. (2004). Bitter Taste Receptors for Saccharin and Acesulfame K. J. Neurosci..

[65] Kawano Y., Kiuchi H., Haraguchi T., Yoshida M., Uchida T., Hanawa T. (2017). Preparation and Evaluation of Physicochemical Propertiesof Isosorbide Gel Composed of Xanthan Gum, Locust Bean Gum and Agar for Improving the Patients Adherence. Int. J. Med. Pharm..

[66] Kadam V.S., Kendre J., Shendarkar G.R., Kadam S.S. (2020). Formulation and Evaluation of Medicated Oral Jelly of Trazadone Hydrochloride. Int. J. Pharm. Sci. Res..

[67] Kobayashi Y., Habara M., Ikezazki H., Chen R., Naito Y., Toko K. (2010). Advanced Taste Sensors Based on Artificial Lipids with Global Selectivity to Basic Taste Qualities and High Correlation to Sensory Scores. Sensors.

[68] Pimenta Pereira P.A., de Souza V.R. (2019). Influence of gelling agent concentration on the characteristics of functional sugar-free guava preserves. Emir. J. Food Agric..

