# Supplementary material for: Evaluation of Physicochemical Properties of a Hydrocolloid-Based Functional Food Fortified with Caulerpa lentillifera: A D-Optimal Design Approach
Source: Gels. 2023 Jun 29;9(7):531. doi: 10.3390/gels9070531 (PMC10379153; doi:10.3390/gels9070531)
Supplement: Supplementary file 1 [file gels-09-00531-s001.zip › gels-2414469-supplementary.pdf]

Supplementary materials

Table S1: The color analysis of the fortified jellies.

Formulation	L*	a*	b*
1	27.71±0.54	-3.56±1.55	10.0±1.70
2	26.83±0.04	-3.25±0.78	6.45±0.87
3	25.78±1.03	-5.28±1.28	10.58±1.96
4	29.34±0.38	-0.79±0.95	11.46±2.57
5	28.93±1.00	0.40±0.31	4.66±0.29
6	29.72±3.08	-0.19±0.29	5.61±0.87
7	31.08±0.74	0.03±0.29	3.48±0.15
8	36.87±1.05	0.18±0.15	2.73±0.40
9	39.48±0.39	-0.13±0.27	5.09±0.15
10	32.57±1.62	-0.04±0.40	4.89±0.51
11	33.03±1.02	-0.22±0.09	4.92±0.18
12	28.85±2.14	-0.39±0.51	4.73±0.49
13	28.14±0.58	0.24±0.02	7.71±0.332
14	37.76±2.23	0.23±0.41	4.78±0.50
15	30.91±1.54	-0.13±0.20	7.59±0.76
16	37.53±1.16	0.14±0.31	6.55±1.97
17	28.64±3.09	-2.52±0.86	3.18±0.96
Control	33.86±4.71	-0.22±0.24	5.15±1.88
Extract	29.87±0.73	0.74±0.55	14.57±1.80
p-values	0.0006	0.000	0.000
